# Parental Assessment of Benefits and of Dangers Determines Children’s Permission to Play Outdoors

**DOI:** 10.3390/ijerph191811467

**Published:** 2022-09-12

**Authors:** Boris Jidovtseff, Florence Pirard, Anne Martin, Paul McCrorie, Andora Vidal, Elodie Pools

**Affiliations:** 1 Department of Sport and Rehabilitation Sciences, Research Unit for a Life-Course Perspective on Health and Education, University of Liège, 4000 Liege, Belgium; 2 Department of Education and Training, Research Unit for a Life-Course Perspective on Health and Education, University of Liège, 4000 Liege, Belgium; 3 MRC/CSO Social and Public Health Sciences Unit, University of Glasgow, Glasgow G3 7HR, UK; 4 Teaching Department, HELMo University College, 4000 Liege, Belgium

**Keywords:** children, outdoor play, risky play, parents, perception, benefit-danger balance, decision-making, photo-based questionnaire

## Abstract

During the early years, children’s outdoor play is dependent on parental supervision. Parents’ perceptions are likely to influence what the child is permitted to do. To better understand the involved mechanisms in parents’ decision making in such contexts, an online photo-based questionnaire was administered. The tool investigates, in different situations, parents and their children’s experience, parents’ perceptions, and permission to play. A total of 417 parents of children aged from 1.5 and 6.0 completed the questionnaire. Results showed that parents, overall, have a positive attitude towards outdoor play. Main concerns were about risk of injury but in most cases, perceived benefits outweigh perceived dangers. “Sawing wood” was the only situation with a negative benefits/dangers balance. A linear regression analysis revealed that permission to play outdoors is based on parental assessment of benefits and dangers. Perceived benefits appeared to have more influence on parental decision than perceived dangers, while perceived competence had only a small influence. The results also showed that parents’ childhood experience of outdoor play was an important determinant for adults’ perceptions, perhaps demonstrating intergenerational concerns, as outdoor play is in decline. To overcome a negative intergenerational effect on children’s outdoor play, interventions and communication should focus on associated benefits.

## 1. Introduction

A growing body of literature highlights that outdoor play is associated with many health, well-being, and developmental benefits for children [1,2,3,4], which often cannot be developed as optimally in an indoor environment [5]. The outdoor environment offers children large spaces and multiple opportunities for discovery that allow them to move, play, experiment, express themselves freely, and to assert their personality [6]. Outdoor play, especially when it takes place in a natural and stimulating environment, has been demonstrated to be effective in improving children’s motor and physical skills [7], body schema, and self-confidence [6]. Moreover, interactions with nature during the early years have been shown to have a positive effect on connectedness with nature and environmentally friendly behaviour in the long term [5,8].

Unfortunately, opportunities for children’s free outdoor play have been greatly reduced in recent decades [9,10,11,12]. This evolution is closely linked to rapid changes in society. There has been a significant increase in children’s participation in supervised activities and an increased parental dependence on travel at the expense of unorganised outdoor activities [13]. Parental availability becomes a key factor in determining opportunities for outdoor play [14]. Moreover, screen time has become an increasingly important part of leisure activities at the expense of time spent outdoors, even in preschool children [15]. Urbanisation and increased traffic have also reduced opportunities for free outdoor play [6], and parents are more reluctant to leave their children unsupervised in a public place [13].

The emphasis on accident prevention for children has been accompanied by high levels of safety measures aimed at zero risk, with important implications for the design of outdoor play spaces [9], regulatory requirements [16], and the behaviour of supervising adults [16,17]. This has effectively led to a safer society supported by statistics showing a decline in child accidents (i.e., traffic accident, falls, drowning, suffocating, etc.) over recent years [1,11]. However, some authors have warned that overly restrictive measures might be counterproductive and may not allow children to develop fully [6,9,18,19]. In Canada and Australia, for instance, many playgrounds are designed to keep children safe rather than to accommodate children’s preferences and developmental needs [9]. A negative consequence of an excessive safety approach is that children encounter less and less challenging situations in their playing environment while they should experience new things, test their limits and adjust their behaviour in response to the situations [20]. There is an emerging body of evidence that highlights this problem while emphasising the developmental value of risk taking in children’s play [6,9,19].

The definition of risk is important yet varies substantially within the literature [21]. In some developmental literature, risk and risk taking often have a negative connotation as they are defined as engagement in behaviours that are associated with a probability of negative outcomes such as loss or injuries [22]. This definition fails to acknowledge that risk-taking behaviours may have positive outcomes and should be considered in the light of the child’s growth and development [22]. A more holistic definition of risk taking involves making a choice within a range of possible actions in which the outcome is uncertain and could be either positive or negative. This is the case when a child takes the decision of engaging in an uncertain, new, and unknown action after having weighted the potential benefits of success against the undesirable consequences of failure. Such challenging play has been widely named “risky play” in the literature and has appeared to be essential in children’s development. Sandseter’s [23] work has established six main categories of risky play that may be accompanied by a risk of accident, but which are of undeniable importance for children’s development: (1) play with great heights; (2) play with high speed; (3) play with dangerous tools; (4) play near dangerous elements; (5) rough-and-tumble play; (6) play where children can disappear or get lost. According to Ball et al. [24], “risky play” is not necessarily a danger that should be avoided but rather a situation that should be managed by the child. When children engage in risky play, they enter what some authors call the ”zone of proximal development”, that is the conceptual space in which learning is most effective [25]. The control that the children have over the situation allows them to progressively increase the level of difficulty of the task [25], contributing to their motor and physical development [7], but also their self-confidence [26,27]. Then, through their experiences, children become familiar with the environment, discover what is dangerous and learn to manage the risks they encounter by developing anti-phobic strategies [28,29,30]. Without exposure to such contexts, children would not have the opportunity to develop such effective strategies for coping with fearful conditions and could therefore avoid engaging in any challenging situations [28].

Researchers who advocate for risky play emphasise that children are naturally attracted to situations that provide sensations and emotions such as swinging, spinning fast, or jumping from a height [29,31]. Children experience excitement and joy when they master a potentially dangerous situation, being fully aware of the fear and risks involved [30].

For many experts, allowing children to engage in risky play will have a beneficial effect on their ability to cope with hazards, both in childhood and later as adults, especially in emergency situations [29,31]. In this sense, exposing children to an acceptable degree of danger, whether on playgrounds [18] or in nature [7], has an educational purpose, which remains poorly recognised. However, it is important to consider the level of danger and to distinguish between injuries according to their severity. Risk taking should not be confused with hazardous child endangerment, which occurs in really dangerous situations that can be fatal and in which children are not aware of the risks in injuries involved [28,32]. According to some authors [28], minor injuries that do not have long-term consequences are among the acceptable consequences associated with a child’s engagement in normal, stimulating activities that are essential for learning and development. Little and Eager [32] revealed that parents generally do not intervene to prevent risky play, but give warnings to children about how to do the activity safely. The role of adults is to avoid any situation, for their children, that is truly dangerous whilst allowing them to engage in play where they can recognise the ability of their children to assess their own risk taking and/or to learn it [28]. This is the case in all six of the above-mentioned categories of risky play, but this is also the case in other contexts which can be encountered by children when playing outdoors. Certain circumstances such as meeting pets or wild animals, discovering edible plants and berries, playing barefoot, and going outside in inclement weather—that are part of the outdoor environment and could also present certain dangers for children [33,34]—are also a concern for parents when children are playing outdoors.

Parental attitudes toward children outdoor play are built on the basis of several correlates such as personal experience, education, sensitivity, socio demographic, environment, or socio-cultural context [6,19,22,35]. It leads to different ways of perceiving things, which may explain why from one person to another, from one country to another, and from one culture to another, the way of looking at outdoor play is sometimes very different [17]. For example, adults’ and children’s attitudes to outdoor weather seem to be strongly influenced by the socio-cultural context [6,17,36]. In many countries, uncomfortable weather conditions such as rain or cold are a major constraint to outdoor activities, whereas in the Nordic countries, parents and childcare professionals seemed to be more concerned about what to wear rather than questioning going outdoors with children [17,36,37]. Several recent studies highlighted cross-cultural differences from one country to another in terms of risky play, but also rules and recommendations [9,38]. Cultural differences are also visible in adapted safety standards [16] and in official programmes and recommendations [39]. For example, Norway officially recognises the benefits of risky outdoor play for children and encourages its practice [19]. In many other countries, the approach is often more protectionist with stricter legislation and safety regulations. Fear of litigation favours absolute safety over the developmental benefits of risky play [17].

Within the same culture, attitudes toward outdoor play can be very different, and vary from one parent to another. Parenting style [14,40], experience of risk taking [41], and feeling of insecurity related to perceived dangers [42] are likely to influence child engagement in risky play. Children’s outdoor play highly depends on an adult’s analysis of the situation and consequent decisions. According to some authors, parents allow their child(ren) to play when the potential benefits outweigh any undesirable consequences [18,22,43,44]. Considering potential benefits and potential negative risks (i.e., dangers) and assessing their balance is considered as a key process in decision making [21]. The “play balance model” developed by Ball et al. [18] for outdoor play also relies on this concept. However, this assessment was never considered in the above-mentioned studies, probably because the benefits are of a different nature than the hazards and therefore cannot be directly compared. Moreover, parental decision making is mostly based on a subjective analysis of the situation rather than on objective criteria. Their response to a risky situation may depend on their personal experience, affective response, and perceived control over the situation [22]. According to the “affect heuristic” theory reported by Slovic et al. [45], the affective dimension related to a singular situation significantly influences perception of associated benefits and dangers. This means that in the same context, the parent who perceives more danger will tend to restrain the child in play, whereas the parent who perceives more benefit will tend to encourage it. It is also likely that attitude toward outdoor play is not only dependent on the situation itself, but also on how parents perceive their child’s competency in the situation. Work of Loprinzi and Trost [46] showed that perception of children’s competence in an activity is related to parental support and therefore may also influence the permission to play outdoors. However, it is still unknown if perception of child competence is directly involved in the parental decision-making process.

The current body of knowledge suggests that parents’ perceptions about outdoor play situations may play a key role in their decision making. However, the various studies that have looked at the impact of parental perceptions on children’s outdoor play opportunities have used very different methodologies [20,40,41,44]. Those papers employing qualitative approaches have used semi-structured interviews to analyse how adult attitudes may influence children’s play [20,41], but were not able to verify the extent to which the different key perceptions (of dangers, benefits, and of child competence) affect parental decision making. Two studies [40,44] used questionnaires to analyse the general attitude of parents to different outdoor play situations. The questions focused on what parents allowed their children to do in different risky play situations but did not ask parents about the perceived benefits and dangers associated with the situation, nor about their child’s perceived competence. Many things remain to be understood about the mechanisms involved in parental decision making in the context of outdoor play.

To date, and to the best of the authors’ knowledge, no study has systematically described parents’ attitudes in different situations of risky outdoor play and no study has proposed a quantitative approach to explore the links between parental experiences and perceptions and children’s permission to play. The purpose of this study was to fill this gap by exploring parental attitudes toward outdoor play and their role in the decision-making mechanisms. To achieve this goal, we therefore decided to develop a photo-based questionnaire that collects information from parents to better understand the decision-making mechanisms in different outdoor play situations in which their child might be involved, and includes: the experience of the parent (PExp) and the child (CExp) in the described situation; perceptions of benefits (PBen) and of dangers (PDang) associated with the situation; parental perception of their child’s competence (PComp) in the play situation; and permission to play (PERM). This tool allows us to meet our two main research objectives. The first aim was to obtain descriptive data about parents’ perceptions and level of permission in different outdoor play situations. The second aim was to investigate how parental perceptions and potential determinants may influence parents’ permission for their child to play outdoors. For the first objective, we assume that parents’ attitudes will vary from one situation to another. However, we expect that there will be sufficient internal consistency across the ten situations, within each dimension, to be represented by an average score; allowing us to investigate the interrelations between the different dimensions. If this is confirmed by our results, we hypothesise that permission to play outside will mainly depend on the perceived benefits and dangers, and on the perceived competence of their child.

## 2. Materials and Methods

### 2.1. Type of Study and Ethical Committee Aproval

This is a cross-sectional study based on a self-administered online questionnaire. The study was funded by the Office of Birth and Childhood (ONE) of the Wallonia & Brussels Federation and approved by the Human and Social Sciences Ethical Committee of the University of Liège, Belgium.

### 2.2. Concept of the Photo-Based Questionnaire

**Figure 1:**
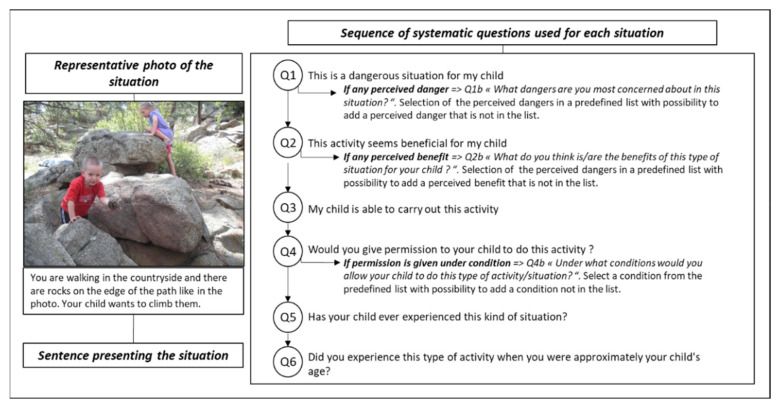
Illustrative representation of the photo-based questionnaire and presentation of the sequence of systematic questions used for each situation. This is an English translation of the original French questionnaire.

To meet the research objectives, it was decided to use a photo-based questionnaire. The concept consists of presenting various outdoor play situations represented by a combination of a sentence describing the context and a picture selected to show what the situation looks like (for an example, see Figure 1). For each photo-based situation, participants were asked to answer questions related to their perceptions, own experience as a child, experience of their child, and permission to play outdoors. The aim of this procedure was to facilitate the expression of responses while limiting the possibilities of confusion or over-interpretation that could be found in a questionnaire without pictures. Although this type of questionnaire is still uncommon, increasingly more scientific work incorporates the use of drawings [47], photos [48], videos [49], or even virtual reality [50] to improve the understanding and representativeness of the situation, thereby reducing the latitude for interpretation. The use of photos associated with a descriptive sentence aimed to create virtual situations that are as credible as possible to allow participants to project themselves into the situation and to facilitate the situation’s representations. It ensures that participants all refer to the same situation (the one described and depicted in the photo) when answering the questions. This approach has three other important advantages: firstly, it allows an explicit and technically easy to use setting for both online and paper questionnaires; secondly, it makes it possible to use the same perception scales (such as perception of benefits, of dangers, of competence) across different situations, these systematic questions allowing analyses across all situations collectively whilst retaining the ability to explore individual situations and to achieve comparisons between situations; and finally, it enables the addressing of situations that have never been experienced by participants.

### 2.3. Age Range, Construction, and Validation of the Questionnaire

The data used in the present study forms part of a larger research project [51] that focuses on children between the ages of 1.5 and 18. In order to develop a photo-based questionnaire adapted to a wide age range, a sequence of questions investigating parental representations of outdoor play situations was developed specifically for three age groups: (i) 1.5 to 6; (ii) 7 to 12; and (iii) 13 to 18 years old. For each age group, specific outdoor play situations were selected to be most relevant to what children experience at their specific age. For this publication, we focus on the 1.5–6-year-old age group because of the importance of early learning and because parental supervision is likely to be higher with young children. Each of the three questionnaires was rigorously developed according to the principles of user-centred design [47] including the respect of questionnaire building rules and the assessment of the usability of the questionnaire. This process took place in three stages: (1) initial construction of the questionnaire; (2) validation by the “think aloud” protocol; and (3) establishing the final form of the questionnaire. Only the procedure for constructing the questionnaire for children aged 1.5 to 6 years will be detailed.

#### 2.3.1. Initial Construction of the Questionnaire

As no validated scientific tool could answer our research questions in a comprehensive and specific way, a photo-based questionnaire was developed tailored to our objectives. The target population was parents of children aged between 1.5 and 6.0 years. To meet our research objectives, the photo-based questionnaire included a selection of outdoor play situations (Table 1) that may be perceived as risky for the children and may require parental approval. Each situation was presented with a combination of a sentence and a picture describing a child in outdoor play (Figure 1).

For each situation, the parents were systematically questioned on the 6 main dimensions covered by the questionnaire: perception of dangers (Q1; PDang); perception of benefits (Q2; PBen); perception of child competence (Q3; PComp); permission to play (Q4; PERM), child’s experience in the presented situation (Q5; CExp); and parental experience as a child (Q6; PExp). Questions were the same for each situation. PDang, PBen, PComp, PERM, and CExp were measured by means of Likert scale; the question on parental experience (PExp) was dichotomous. Three questions had a filter function (Q1, Q2 and Q4), enabling opening of sub-questions for a more descriptive analysis. When parents perceived a situation as dangerous, they were asked the following additional question: “What danger(s) are you most concerned about in this situation?” (Q1b). They were asked to select potential perceived dangers from a predefined list of answers. At the end of this list, they were given the opportunity to add any other perceived danger that was not included in the predefined list. A similar approach was used for the perception of benefits “What do you think is/are the benefit(s) of this type of situation for your child?” (Q2b) and for permission to play “Under what conditions would you allow your child to do this type of activity/situation?” (Q4b). In each of these sub-questions, pre-defined lists were established by the researchers and individualised to each situation (see Supplementary Materials).

The initial version of the photo-based questionnaire included 12 situations. Eight of them were associated with the main risky play categories presented by Sandseter et al. [23]. In addition to these categories, we decided to add four other contexts not included in Sandseter’s list: meeting animals [17,52,53,54], discovering edible and non-edible plants and berries [55], playing barefoot [56], and playing in inclement weather conditions [36,37]. All these situations can be associated with outdoor play, have educational interests and might be subject to parental permission as they can be associated with real or perceived dangers such as the risk of being bitten [33,54], poisoned [34], burned [57], or getting sick, respectively [58]. The choice of these four situations, specifically, remains debatable because they do not all present the same level of danger and cannot be associated in the same way with the concept of risky play presented by Sanseter [31]. Nevertheless, they are part of the reality of children’s outdoor play and might be subject to the parents’ permission to play.

The selection of the photos illustrating each of the situations appeared to be a very important stage in the construction of the questionnaire, since what is illustrated in the photo is likely to influence interpretation. One of the challenges was to find and select photos that were as close as possible to the reality that we wanted to present to the parents. The children had to correspond to the target age group, and the situation illustrated by the photo had to combine elements that could be perceived as beneficial but also as risky by parents. To avoid floor or ceiling effects, it was important to identify contexts that would not be perceived by most parents as too risky or as not risky at all. The selected photos also had to meet several criteria: (a) to illustrate the situation described in an understandable way; (b) to be credible for children aged 1.5–6.0 years; (c) to include both girls and boys; (d) to be free of copyright or have the rights to use them.

Parents who had more than one child in the target age category were asked to select one child at random and complete the questionnaire in reference to the chosen child. Parents were asked to consider each situation like it was their child in the picture (see Figure 1 for an example). They were then asked to answer the questions even if they had never encountered the situation and even if the situation did not correspond perfectly to their reality (for example, the child had never ridden a balance bike because he or she was too young or because there was no balance bike at home). Using a photo-based situation and asking parents to project themselves into it with their own child aimed to help parents giving a response that was representative of their perceptions, even when the situation had never been experienced.

#### 2.3.2. Think Aloud Technique Validation

To validate the understanding of the questionnaire and to obtain critical feedback on the content, we administered the photo-based questionnaire to five parents using the “think aloud” protocol (TAP). The parents came from different socio-economic backgrounds and lived in contrasting urban contexts. The TAP consists of administering the questionnaires by asking the person to verbalise whatever crosses their mind during the task performance [59]. The audio recordings were then transcribed and analysed. Comments considered as relevant and allowing for the improvement of the questionnaire were retained in order to: (1) improve the correct understanding of all the questions and answers; (2) remove inconsistencies and redundancies in the questionnaire; (3) complete the list of pre-established answers for questions Q1b, Q2b, and Q4b; (4) confirm the credibility of the situations and photos proposed; and (5) verify the time taken by the parents to answer the questionnaire. The photo-based questionnaire was considered by parents participating in the TAP as very understandable, complete and relevant, but was also reported to be quite long. Consequently, the major improvement consisted in removing two situations to shorten the questionnaire. Situations were removed because of conceptual similarities with other situations.

#### 2.3.3. Final Version of the Questionnaire

The validated version of the photo-based questionnaire included the structure presented in Figure 1 and the 10 selected situations are listed in Table 1. Final questions, answers, and scores associated with the main dimensions are reported in Table 2.

Additional information on level of education (5-level scale from primary education to master’s degree or equivalent), income (5-level scale from <EUR 1250 monthly family income to >EUR 10,000 monthly family income), gender of the parent (male = 0; female: 1), and age of the child was also asked in the survey to complete the analysis.

### 2.4. Dissemination of the Questionnaire

The questionnaire was fully encoded on the LimeSurvey platform (LimeSurvey GmbH, Hamburg, Germany). Several means were utilised to disseminate the online questionnaire as widely as possible and to obtain a maximum number of responses. Dissemination was carried out on the Office of Birth and Childhood website, via social networks, via newsletters from child-related associations, and via a network of parents who had already participated in a previous survey [51]. All French-speaking parents living in Belgium and having at least one child between 1.5 and 6.0 years old were eligible to participate in the survey. No exclusion criteria were applied to children, meaning that parents could also complete the questionnaire if their child had a physical, sensory, intellectual, or learning disability. Data was collected between June and October 2019.

### 2.5. Data Processing and Statistical Analysis

Statistical processing was carried out using Excel (Microsoft, USA) and Statistica (Version 13.2, TIBCO Software Inc., Palo Alto, CA, USA). Firstly, the characteristics of the respondents (gender, age of the parent, level of education, and level of income) were analysed by means of frequencies and averages. Then, each situation was described through (i) average scores obtained for each main question and; (ii) the identification of the main perceived benefits, dangers, and criteria of permission that were selected. Only the most frequently cited elements and those cited by at least 50% of parents were reported in the results. Cronbach’s alpha and McDonald’s omega were used to assess the reliability of the 6 main dimensions and a respondent average score for each dimension was computed across the 10 situations. McDonald’s omegas were specifically computed with the R package MBESS [60] and reliability estimates were interpreted according to Kalkbrenner [61]. Means and standard deviations of these respondent average scores are presented in Table 2. Benefits/dangers balance (BDB) was calculated for each participant and each situation as the difference between PBen and PDang scores.

Pearson coefficient was used to determine correlations between variables. Strength of the correlation was interpreted according to Dancey and Reidy [62]: perfect for r = 1; strong for 0.9–0.7; moderate for 0.69–0.4; weak for 0.39–0.1 and zero for r = 0. A multiple univariate linear regression approach was then used to investigate theoretical models. We hypothesised that parental perceptions (PBen, PDang and PComp) were predictor variables explaining the level of parental permission to play outdoors (PERM). We also hypothesised that personal factors such as PExp, CExp, and children age (CAge) might affect parental perceptions. Therefore, it was decided to conduct the multiple univariate linear regression analyses in two steps. In the first step, personal variables were used to explain each parental perception separately (Equations (1)–(3)). The second step aimed to determine how PBen, PDang, and Pcomp may explain PERM (Equation (4)). In this second analysis, personal factors were also included as covariates in the model, to investigate if they could also predict permission to play.

(1) $${\hat{\mathrm{PBen}}}_{i}\;=\;b_{0}\;+\;b_{1}{*\mathrm{CAge}}_{i}\;+\;b_{2}{*\mathrm{PExp}}_{i}\;+\;b_{3}{*\mathrm{CExp}}_{i}$$

(2) $${\hat{\mathrm{PDang}}}_{i}\;=\;b_{0}\;+\;b_{1}{*\mathrm{CAge}}_{i}\;+\;b_{2}{*\mathrm{PExp}}_{i}\;+\;b_{3}{*\mathrm{CExp}}_{i}$$

(3) $${\hat{\mathrm{PComp}}}_{i}\;=\;b_{0}\;+\;b_{1}{*\mathrm{CAge}}_{i}\;+\;b_{2}{*\mathrm{PExp}}_{i}\;+\;b_{3}{*\mathrm{CExp}}_{i}$$

(4) $${\hat{\mathrm{PERM}}}_{i}\;=\;b_{0}\;+\;b_{1}{*\mathrm{CAge}}_{i}\;+\;b_{2}{*\mathrm{PExp}}_{i}\;+\;b_{3}{*\mathrm{CExp}}_{i}\;+\;{\;b}_{4}{*\mathrm{PBen}}_{i}\;+\;b_{5}{*\mathrm{PDang}}_{i}\;+\;b_{6}{*\;\mathrm{PComp}}_{i}$$

## 3. Results

### 3.1. Participants

Of the 536 participants who started the self-administered photo-based online questionnaire, 417 completed all questions (completion rate of 78%). Only complete questionnaires were retained for analysis. Participants were mostly female (88%) with an average age of 34.8 (standard deviation = 5.6) years. The average age of the children for whom the questionnaire was completed was 3.7 (standard deviation = 1.5) years. Regarding income and education categories, the majority of respondents were from middle to high income families (69%) and most of them stated that they had completed higher education (80%).

### 3.2. Descriptive Analysis of the 10 Situations

Table 3 shows the average scores (with standard deviation in brackets) for each of the six investigated dimensions as well as for BDB in each situation. It also lists the main perceived benefits, perceived dangers and conditions of permission to play that were the selected by the parents from the predefined lists determined for each situation.

The situations perceived by the parents as the most beneficial were “playing in the woods” (mean = 2.4) and “running barefoot” (mean = 2.4), while the ones perceived as least beneficial were “sawing wood” (mean = 1.6) and “petting a dog” (mean = 1.9). The analysis of the main perceived benefits (benefits selected by more than 50% of the parents) showed important differences between situations. “Climbing on a rock”, “riding a balance bike”, and “playing in the woods” were associated with a large number of benefits. Child’s enjoyment and the discovery of new sensations were considered benefits in 7 out of 10 situations. Parents stated that 6 out of 10 situations were beneficial for discovering the environment and/or being in contact with nature, but also allowed for knowing one’s limits and managing risks and dangers. Developing motor skills, agility, and balance was considered a main benefit in 5 out of 10 situations, especially in “climbing on a rock” and “riding a balance bike” situations. Self-confidence was also reported to be a beneficial outcome related to 5 out of 10 situations. The activities “sawing wood” and “eating berries” were perceived as beneficial only for their specific context, i.e., the development of a functional and/or practical activity in the first case and discovering the environment and/or being in contact with nature and the discovery of new sensations in the second case.

The situations perceived as the most dangerous by parents were “sawing wood” (2.0 ± 0.9), “petting a dog” (mean = 1.6), “playing on the edge of a pond” (mean = 1.6), and “climbing a rock” (mean = 1.5). Conversely, “running barefoot” (mean = 0.9), and especially “playing in the rain” (mean = 0.4), were not perceived as dangerous activities. The analysis of the main perceived risks (risks selected by more than 50% of the parents) shows that in 6 out of 10 situations the main fear of parents was that the child would be physically harmed (falling, getting hurt, getting cut, being bitten, etc.). The dangers perceived by parents were very specific to the situation. For example, for “playing in the woods”, the main perceived danger was that the child would get lost. For “eating berries”, the main perceived danger was that the child could eat an inedible berry. In two contexts, “sawing wood” and “playing by a pond”, they also feared that their child could repeat this behaviour when unsupervised. Benefits/dangers balance was positive in all situations except for the “sawing wood” situation.

Parents perceived their child as relatively competent in 8 of the 10 situations. The two situations in which parents considered their children as less competent were “sawing wood” (mean = 1.5) and “eating berries” (mean = 0.8). These two activities were also the ones most restricted by parents with PERM mean scores < 1.0. In all situations, the majority of parents allowed the child to do what was depicted, but under specific conditions (from 54% to 84% of respondents selected the related response “yes, under condition” in Q4; according to the situation). The main conditions for permission also appeared to be specific to each situation and were linked to adult supervision (6 out of 10 situations), suitable equipment (“riding a balance bike” and “playing in the rain”), an environment perceived as not very dangerous (“climbing a rock”, “riding a balance bike”, and “running barefoot”), or to a context perceived as non-aggressive (“playing with wooden swords”).

The results show a certain parallelism between the experiences of parents and children. For both populations, “sawing wood” was by far the least experienced situation, whereas “running barefoot” and “petting a dog” were situations experienced by almost all parents and children (>86%).

### 3.3. Internal Consistency of Main Dimensions

Cronbach’s alpha and McDonald’s omega were used to assess the reliability of the 6 main dimensions (PExp, CExp, PDang, PBen, PComp, and PERM). Coefficients provide evidence of acceptable (alphas and omegas > 0.7) to strong (for the perceived benefits’ omega, above 0.8) reliability for all scales.

### 3.4. Correlation and Linear Regression Analyses

Table 4 presents the Pearson coefficients of correlations between parental perceptions, permission to play, child age, and parent and child experiences. All perceptions were correlated with each other and presented moderate to strong correlation with PERM. PBen presents moderate to high correlation with experience (PExp and CExp), other perceptions and permission to play but is not related to CAge. PDang is negatively correlated with the other variables: the highest correlations can be observed with PBen (r = −0.55; *p* < 0.001) and PERM (r = −0.60; *p* < 0.001). In other words, a higher level of perception of danger is associated with less perceived benefits and a lower score of authorisation; it is also, to a lower extent, associated with a lower perceived competence of the child and less parental and children experiences. PComp presented the highest correlations with PBen (r = 0.66; *p* < 0.001) and CExp (r = 0.67; *p* < 0.001) and was also moderately correlated with PERM (r = 0.58; *p* < 0.001), CAge (r = 0.40; *p* < 0.001), and PDang (r = −0.41; *p* < 0.001). Moreover, PExp is positively related with CExp (r = 0.52; *p* < 0.001), meaning that experience when parents were a child is correlated with child-reported experience. Finally, the parental perceptions of benefits (r = 0.75), of danger (r = −0.60), and of competence (r = 0.58) are associated with PERM: parents are thus more susceptible to authorise outdoor play when the perceived benefits are higher, when they believe that their child is able to do the activity, and when they perceive less dangers.

Multiple linear regression analyses used to predict the three perceptions are presented in Table 5 accordingly to equations 1 to 3. All three models were significant in explaining the dependant variable’s variability (*p* < 0.001). Adjusted R^2^ is lower for PDang (R^2^ = 0.13) than for PBen (R^2^ = 0.35) and PComp (R^2^ = 0.47). Results showed that the PDang score decreased when CExp and PExp increased, while CAge appeared not to influence PDang under control of child and parent experiences. When PExp increases by one point, PDang decreases by −0.19 points (*p* < 0.001) and a one-point increase in CExp is associated with a decrease of −0.40 (*p* < 0.001) in PDang score, other independent variables being held constant. It means that parental and child experience in the situations were both associated with a lower level of danger perception, and conversely a low level of experience was associated with a high danger perception. PBen was significantly (*p* < 0.001) associated with the three predictive variables included in the model (CAge, CExp, and PExp). However, the analysis of standardised beta coefficients revealed that CExp (b* = 0.53) had more influence on PBen than PExp (b* = 0.17) and CAge (b* = −0.16).

The results of the multiple regression analysis showed that PComp was significantly predicted by CAge and CExp, but not by PExp. Beta coefficients revealed that CExp appears to be the more influential variable: as CExp increases by one point, PComp increases by 0.41 points (*p* < 0.001). In other words, under control of parental experience and child age, the parents’ perceived competence of their child increases as their child’s experience increases.

Table 6 presents the results of the linear regression between explanatory variables and PERM (Equation (4)). The model has a high level of prediction (adjusted R^2^ = 0.62). PBen (b* = 0.52; *p* < 0.001) and PDang (b* = −0.27; *p* < 0.001) were the only two variables reaching significance. Unsurprisingly, PBen and PDang have opposite associations on PERM. However, the magnitude of the association with PBen appeared to be double in comparison with PDang, signifying that PBen had a stronger association with PERM than PDang. PComp was significant at *p* < 0.1 but had a small effect on PERM (b* = 0.09) under control of the other predictors included in the model, meaning that the permission to play was mainly related to PBen and PDang. Pexp, CExp, and CAge were not significantly related to PERM, other predictors held constant.

## 4. Discussion

The first aim of this research was to obtain descriptive data about parent’s perceptions and level of permission to play in different outdoor situations that could concern children aged 1.5 to 6 years. Results summarised in Table 3 confirm our hypothesis that parents’ attitude toward outdoor play would be relatively consistent but not completely uniform from one situation to another. Each of the ten situations have specificities in terms of perceived benefits, perceived dangers, perceived competences, and conditions for playing. Parents perceived benefits for their child in most of the situations; these results are in line with several studies where adults (parents or professionals) generally display a positive attitude towards outdoor play with young children [20,22,43,44,63,64]. The fact that parents associated several benefits with situations varying between “climbing on a rock”, “riding a balance bike”, or “playing in the woods “, “running barefoot” or “playing on the edge of a pond” confirmed that they recognised the multidimensional value of outdoor play [22,43].

Participating parents considered most of the depicted outdoor situations as a source of fun for their children but also as suitable contexts for environmental discovery and global development. Half of the proposed situations were reported to be beneficial for the children for discovering their environment and to be in contact with nature. That point is very important as nature-based outdoor play is supposed to have positive effects on children’s connectedness with nature and on their acquisition of environmentally friendly behaviour [4,5,8,65]. Half of the situations were recognised by parents as being beneficial for their children in the discovery of personal limits and in the management of risks and dangers. Learning to manage risk is precisely a major outcome of any outdoor risky play [6,9,23,31] and it helps to develop anti-phobic strategies that can be used later for coping with fearful situations [28]. Risky play is also reported to improve motor skills [7,43], self-confidence [6], autonomy [5,66], and to explore senses [5,67]. All these benefits were largely mentioned by the parents who seemed to recognise the benefits of situations that have a high physical developmental potential for children such as “climbing on a rock”, “riding a balance bike” and “playing in the woods”. However, Sandseter and Kennair [28] stated that children do not engage in a situation for the benefits it provides but for the enjoyment they find in it. This notion is very important as it is central to motivational processes [68]. Nevertheless, as it speaks to the data from this study, it is parental perceptions of benefits that are being interpreted, not the children’s, per se. So, we cannot make any statement about why the children like to take part in risky scenarios; we can only interpret why the parents think it may be beneficial. It is interesting to underline that some situations were associated by parents with limited but specific benefits. It was the case with the “sawing wood” situation, where only the “development of a functional task” was recognised as a benefit, and with the “eating berries” situation, which was associated with the “discovery of the nature and environment” and the “discovery of new sensations” benefits. This argues that, according to parents, outdoor play offers benefits that are not equal but reveals a diversity and complementarity that is interesting for the development of the child and for his or her integration in the living environment.

The perception of danger also varied greatly from one situation to another. For example, playing in the rain does not seem to be associated with great danger (mainly the risk of getting sick) whereas sawing was perceived as much more so (cutting oneself). In fact, the perception of dangers appeared to be highest in outdoor play situations with potential of harmful consequences such as cutting skin when “sawing wood”, slipping and hurting when “climbing a rock”, being bitten by a dog when “petting a dog”, and falling and drowning when “playing on the edge of a pond”. The “sawing wood” situation presented the highest perception of danger and was the only one to have unfavourable negative benefit/danger balance. In fact, the overall results showed that this situation was perceived very differently by parents than the other outdoor play situations. The use of real tools by children aged 1.5 to 6.0 years old might be surprising but falls into the categories of risky play proposed by Sandseter [23]. Preschoolers in Norway are regularly involved in playing with real tools such as knives, screwdrivers, saws, and hammers. It was pointed out that although adults perceived these activities as risky, they allowed them while closely supervising the children [23]. According to the author, “play with dangerous tools” is probably the category perceived as most hazardous from an adult point of view, while the children were more inclined to feel that this was only an exciting activity. In such activities it is important for adults to supervise the children very closely and give constant advice on how to hold and use the tool correctly to avoid any risk of accident. Sandseter [23] admits that the use of dangerous tools is a practice that might be specific to Scandinavian countries, particularly Norway, whilst probably less common in other cultures—which is the case in Wallonia & Brussels Federation, the population specific to the current study. Cultural and educational particularities are known to influence adults’ beliefs and practices [6,19,22]. Such cross-cultural differences in risky play, rules, and recommendations have been previously reported [9,38] and deserve further research. Parents’ beliefs about the risks and safety of an activity are likely to influence the level of positive health risk-taking tasks considered as acceptable for a child [32] and may explain why in the present study sawing wood is the outdoor activity less likely to be allowed by parents.

Petting a dog is a situation associated with a moderate level of PBen and PDang and appeared to be the second most restricted situation according to parents. While the importance of contact with animals during early childhood education has been highlighted by research [52,53], young children are at greater risk of dog bite incidents, especially because they have a poorer understanding of dog behaviour and signalling [69]. Safe interaction between children and animals cannot be guaranteed, especially with unfamiliar dogs and adult supervision appears essential. Our results show that it was one of the situations with the highest perception of danger, and for the parents who stated that they would only allow their child to pet the dog under conditions, the main prerequisite was the owner of the dog to be present and to hold the dog on a leash. The parents’ fears are related to the dog’s behaviour and to their child’s behaviour. Even if educational programmes have significantly improved 5-year-old children’s interpretation of dogs’ behavioural signals [70], caution should always be recommended in this age group as a child’s and dog’s behaviour can be unexpected.

Many parents were concerned by the “Playing at the edge of a pond” situation while recognising that it was a fun and beneficial activity for their child. The parents mainly feared their child could fall in the water and could repeat the behaviour alone. Risk of falling into the water is low but real [54], especially for the youngest children who do not yet have sufficient body control or an understanding of the danger that the aquatic environment can represent. Drowning incidents, although rare, remain one of the most important causes of children mortality and many incidents are related to a failure to supervise children [71]. Playing in and around water is a stimulating activity that is highly valued by children, but one that cannot be without careful parental supervision, especially in the age group covered by our study. In our sample, parents who would allow their children to play at the edge of a pond under conditions were mainly concerned by supervising them; more broadly, the results presented in this paper signify the importance of parental supervision of their children during outdoor play, considered in 6 out of 10 situations, as a condition that determined what the child was allowed to do. These results are in accordance with many authors recognising that a secured environment and adult supervision are important conditions when children are playing outdoors, especially in the early years [1,20,72]. However, control and supervision of the activity to ensure safety should be adjusted to the context in such a way that children may continue to play with spontaneity, freedom, and autonomy, which are considered valuable for child development [73].

In the “eating berries” situation, parents were concerned about the risk to pick inedible berries and specified, in most of the cases, that adult verification was necessary. This situation, which was one of the less permitted by parents, also presented the lowest perceived competence score, meaning that parents considered that their child was not competent in recognising edible berries. It is known that a parent’s perception of their child’s competence may influence their support for autonomy and play [8,59] and this could be the case in this context. Children’s ability to recognise toxic and non-toxic fruit has been reported to be low and younger children were more at risk to consume toxic fruits [34]. The cautiousness of parents in this situation is consistent with children’s low knowledge of the plants described in the literature [34]. This situation shows, once again, that in the specific age group covered by our questionnaire, adult supervision is considered as very important in many circumstances. Previous research studies have also demonstrated that children’s abilities to distinguish toxic and non-toxic plants did not improve with age [34], underlining a lack of educational programs on that topic. This underlines that parents should not limit their role to supervision but should also ensure education when accompanying their child outside. In this context, it means accompanying the child in the process of identifying edible berries while informing about the potential dangers related to misidentification and the ingestion of toxic plants and berries found in nature.

Playing in the rain is an interesting outdoor play context which is known to be influenced by the cultural and educational context [17,36,37]. Our results showed that parents perceived this situation as beneficial and not very risky, and mainly conditioned it on suitable clothing. To get sick is perceived as the main risk associated with playing in the rain as previously reported [58]. The very positive attitude of parents toward this situation contrasts with previous research in the same population [51], or in other Western countries [63,64,74], that were reporting rain to be an important barrier to outdoor play and education. However, attitude is not a guarantee of practice, and our study did not measure the frequency of outdoor play practice. Moreover, results of this study showed that the “playing in the rain” situation is, by far, the scenario with the highest benefits/danger balance while its permission score was not higher than other situations perceived as more dangerous (e.g., “climbing on a rock”). It seems that for the situation “playing in the rain” other determinants related to cultural and/or climate specificities may be more influent. It was also surprising to see how parents reported a very positive attitude toward “running barefoot”, while in Belgium a very large majority of young children are playing shod when outside. It is likely that the differences in practice observed between countries concerning these two contexts [17,36,37,75] are more related to habits, cross-cultural and climate differences than to differences in the benefits/danger balance.

While it is clear from our analysis that each situation is particular and that permission to play seems to be conditioned in a specific way, some trends appear and can be highlighted in the parental perceptions and authorisation to play outside. Investigating relationships between parental perceptions and children’s permission (PERM) to play outdoors was one of the main purposes of the present study. While the correlation analysis confirmed that perceptions of benefits, of dangers, and of child competencies were all significantly correlated with PERM, the multiple linear regression highlighted that PBen and PDang were the most significant predictors of PERM. Contrary to our initial hypothesis, it seems that PComp has a small influence on parents’ decision making once controlled for the perceptions of danger and of benefits. Our results also showed that PComp presented high correlations with CExp and PBen and further research is needed to better understand the exact role of perceived competence in parental decision making and relationships with other perceptions and experience. One major finding of the present research is that the permission to play outdoors is mostly affected by the perceptions of benefits and of dangers, and that the assessment of the benefit/danger balance thus may play a key role in parental decisions. These results are in accordance with previous authors [43] claiming that parents let their children play when they consider that benefits outweigh the dangers associated with this activity. This is also confirming the relevance of the “play balance model” developed by Ball et al. [18].

Interestingly, our results from the linear regression and the correlation analyses indicated that PERM has a stronger association with PBen than with PDang. This result may be counterintuitive as in modern society, there is a growing focus on the safety of the children in order to avoid injuries and hazards [28]. However, this result is in alignment with the sequential decision-making scheme described by Vlek [21], in which the perception of benefits plays a key role. According to this model, a situation without any significant perceived benefits is directly rejected while the assessment of the benefit/danger balance is considered on the condition that important benefits are perceived. This means that, in the context of outdoor play, children would be allowed to engage in an activity only when parents perceive it as beneficial. Conversely, a high perception of danger could be a significant barrier to an activity, even if it has many benefits. Perceptions appear to be sensitive levers that can influence parents’ attitudes towards outdoor play. These perceptions are influenced by experiences, knowledge, but also by emotions. The “affect heuristic” theory reported by Slovic et al. [45] shows that the affective dimension related to a singular situation influences the perception of dangers and benefits inherent to this situation. According to this theory, providing information about benefits should change perception of dangers, and vice versa. Therefore, communication centred exclusively on the dangers of an activity will have a negative influence on the representation of outdoor play activities. On the other hand, communication which highlights the benefits of outdoor play, while recognising dangers, offers more complete perception of the situation and should help parents to make an informed decision. Emphasising the benefits of outdoor play and contextualising the potential dangers and the risk of their occurrence, while ensuring safety recommendations, should have a positive impact on perceptions and enable a more appropriate support for children in their play. The development of communication tools and campaigns aimed at parents could therefore help to improve support for children in their outdoor play and in their learning to manage risk and danger [28,36,76]. A communication campaign on this topic was launched in 2017 in the Netherlands with the aim of changing the risk perceptions of parents and professionals, including through a document entitled “We protect children from big risks and teach them to manage small risks” [77]. Communicating and offering such advice on preventive attitudes to limit the risk of accidents while promoting the multiple benefits of outdoor play might prove to be useful for parents and would help increase children’s outdoor play opportunities.

It was also interesting to explore, through our global analysis, how parental perceptions were influenced by personal factors such as variables related to parental childhood experience, child experience and children‘s age. An important result that emerged from this analysis was the significant impact of parental experience on the perception of benefits and dangers. These results are consistent with the critical place of personal experience in the construction of representations, especially in the outdoor play context [6,19,22,63]. Particular attention should be directed to the negative relationship observed between PExp and PDang as it means that parental perception of dangers is higher when they themselves have had little experience of the proposed situations as a child. Childhood and adult experience with outdoor play contribute to develop environmental sensitivity which is defined as “the predisposition to take an interest in learning about the environment, feeling concern for it, and acting to conserve it, on the basis of formative experiences” [78]. Such “environmental sensitivity” may play a key role in adults perceptions on children’s outdoor play [22,78]. Our results also showed a moderate positive relationship between PExp and CExp. These results taken together raise the question of the intergenerational consequences that could result from the reduction in practice and time spent outdoors reported in several studies [9,10,12]. If the parents-to-be have less experience and this low experience increases the perception of danger, this results in fewer opportunities for children’s outdoor play. Thus, encouraging outdoor play from early years appears to be of great importance to break any potential vicious circle. This is particularly true because personal outdoor experience during childhood may play a key role in life-course development of individual environmental sensitivity and could therefore affect adults’ decision making on children’s outdoor play and educational practices [22,78].

### 4.1. Strengths of the Study

One of the strengths of this study is that the original and innovative photo-based questionnaire allowed for a complementary approach, with, on the one hand, a descriptive analysis of the different outdoor play situations, and on the other hand, an analysis of the relationships between experience, perceptions, and parental decision making.

An added value is that this study is the first to investigate the influence of the parental perceptions of benefits, of dangers, and of competences on their decision to authorise outdoor play. The results show that parents seem to assess the benefit/danger balance before giving permission to play outdoors.

One additional strength of the present study is its ability to question the parents on specific situations, even when they have not been experienced by all the respondents. Such an approach supports perspectives of research in the field of representations and decision making in play or educational situations that question the perceptions of benefits and of dangers.

### 4.2. Limitations and Perspectives

Although the methodological approach used appears promising and has been rigorously developed according to the principles of user-centred design [47], it nevertheless requires further studies which should focus on analysing its psychometric properties. Further research could use structural equation modelling to deepen our understanding of the measurement models of parental perceptions and authorisation and to further analyse the relationships between these variables. Certain points for improvement appeared in the course of this study. For example, we noted that the scales used to measure PERM and PExp offer a limited number of possible responses (three for PERM and two for PExp) that could affect sensitivity and should be improved.

Another element that should be given special attention is the selection of the photos that need to be in line with the study population. For instance, the high perception of dangers associated with the “sawing wood” situation may be partly explained by the photo itself. It seems that parents perceived this situation as too risky compared to what children of this age usually do, and an adjustment of the situation and/or the photo could be considered in order to correspond more closely to the reality encountered by children in this age group. In this type of questionnaire, the photo is part of the question and can really influence the results. Furthermore, our survey was aimed at a fairly broad age range (1.5 to 6 years). While handling a saw is a task that can be done with a 6-year-old child, it may be a less acceptable task with a 2-year-old. This issue highlights the importance of proposing photo situations that should correspond as much as possible to the characteristics of the children and perhaps the age range targeted by this study was too wide for an identical series of pictures. The extent to which the adjustment between each of the proposed situations and the age of the child for whom the parents responded to the questionnaire should be further investigated, as it may have an impact on the responses to the questionnaire. The same point can also be made for those parents of children with physical, sensory, learning, or intellectual disabilities, for whom the proposed situations could be less adapted. Further research should be carried out to define more precisely how to conduct the selection of photos according to the characteristics of the target population and should also verify to what extent the selected photos and situations can be used for different age groups as well as for children with disabilities.

The mode of dissemination of the questionnaire used in this study does not allow for a representative sample of the study population. In comparison with the demographic and socio-economic data available for the population studied (Wallonia & Brussels Federation), we have an over-representation of mothers in relation to fathers and the respondents were more likely to come from a privileged socio-economic background. This over-representation of mothers is regularly described in surveys concerning children [43,79]. In future studies, it would be useful to focus on under-represented populations, particularly fathers as well as socially disadvantaged families.

In addition, as these are voluntary and self-administered questionnaires, the sample is likely to include respondents who feel concerned by the subject matter of the survey. The opinions and representations of those less concerned by outdoor play and/or by young children’s education could differ and may lead to bias.

Finally, it is likely that results of the present study were influenced by the socio-cultural context and cannot be generalised to other countries and cultures. This supports the need for more cross-cultural studies and the use of a photo-based questionnaire like the one developed and is an opportunity for further scientific exploration.

## 5. Conclusions

The photo-based questionnaire developed and used in the present study has enabled a better understanding of the parental attitudes towards ten outdoor play situations and their role in the decision-making mechanisms. In agreement with our first hypothesis, the results showed that attitude toward outdoor play varies from one situation to another. Parents have a positive overall attitude towards outdoor play and “sawing a wood” was the only situation where perceived dangers outweighed perceived benefits. The results also suggested that parents recognise the multi-dimensional interest of outdoor play situations which are considered as a source of fun for their children, but also as a source of development and learning. Main parental concerns were related to the safety of their child. One of the main results of the study is that perceived benefits and perceived dangers appeared to be the main predictive variables of permission to play outdoor, confirming that the assessment of the benefits/danger balance plays a key role in the process. Interestingly, perceived benefits appear to be more influential on parental decisions than perceived dangers. Contrary to our initial hypothesis, PComp seems to have a small influence on parents’ decision making. One practical consequence is that if we want to support children’s outdoor play, we must first highlight the associated benefits to parents. Our results invite the development of communication tools and campaigns on outdoor play, informing parents on the benefits, potential hazards, and preventive attitudes so that they can accompany their child and make informed choices. Finally, the linear regression analysis underlines that parent experience when they were a child and child experience are significant predictors of perceptions. This result raises the question of the intergenerational negative consequences that could result from the reduction in practice and time spent outdoors. Thus, encouraging outdoor play from early years appears to be of great importance to break any potential vicious circle. This is particularly true because personal outdoor experience during childhood may play a key role in life-course development of individual environmental sensitivity, which is a major issue for future generations.

## Figures and Tables

**Table 1:** List of situations and associated risk categories in the photo-based questionnaire.

	Situations	Risk Categories
1	Climbing on a rock	Play with great heights *
2	Riding a balance bike	Play with high speed *
3	Sawing wood	Play with dangerous tool *
4	Playing on the edge of a pond	Play near dangerous element *
5	Swordplaying with woods	Rough-and-tumble play *
6	Playing in the woods	Play where children can disappear or get lost *
7	Playing in the rain	Play in inclement weather condition
8	Petting a dog	Meeting animals
9	Eating berries	Discovering plants and berries
10	Running barefoot	Play barefoot

* Risk categories from Sandseter [23].

**Table 2:** For each dimension of the photo-based questionnaire, summary of the response scale and means, standard deviations, and reliability of the average scores (across situations).

		Answers and Scores				Mean Scores (M), Standard Deviation (SD) and Reliability			
Measured Dimensions	Questions	0	1	2	3	M	SD	Cronbach’s Alpha	McDonald’s Omega
Perceived benefits (PBen)	This activity seems beneficial for my child	Strongly disagree	Rather disagree	Rather agree	Completely agree	2.12	0.51	0.83	0.83
Perceived dangers (PDang)	This is a dangerous situation for my child	Strongly disagree	Rather disagree	Rather agree	Completely agree	1.29	0.47	0.74	0.74
Perceived competence (PComp)	My child is able to carry out this activity.	Strongly disagree	Rather disagree	Rather agree	Completely agree	2.13	0.43	0.73	0.74
Permission to play (PERM)	Would you give permission to your child to do this activity?	No	Yes, under condition	Yes, without condition		1.06	0.28	0.73	0.74
Child experience (CExp)	Has your child ever experienced this kind of situation?	No	Probably not	Probably yes	Yes	2.1	0.61	0.78	0.79
Parent experience (PExp)	Did you experience this type of activity when you were approximately your child’s age?	No	Yes			0.74	0.22	0.72	0.72

**Table 3:** Descriptive analysis (mean score (standard deviation)) for perceived benefits (Pben), perceived dangers (Pdang), benefits/dangers balance (BDB), perceived competences (PComp), permission to play (PERM), child experience (CExp), parent experience (PExp), and main benefits, dangers, and permission conditions reported by parents.

	PBen			PDang	BDB	PComp	PERM		CExp	PExp
Situations	Score/3	Main Benefits *	Score/3	Main Dangers *	PBEn–PDang	Score/3	Score/2	Conditions of Permission *	Score/3	Score/1
Climbing on a rock	2.2 (0.8)	MAB. MRD. SCF. NAT. FUN. DAU. SSS. IFF	1.5 (0.8)	To slip and hurt	0.7 (1.3)	2.1 (0.8)	1.2 (0.5)	To be accompanied by an adult Rock is not hazardous	2.2 (1.1)	0.7 (0.4)
Riding a balance bike	2.2 (0.7)	MAB. MRD. FUN. SCF. DAU. IFF. SSS	1.3 (0.8)	To fall and hurt To have an accident. hit something or someone	0.9 (1.3)	2.1 (0.9)	1.1 (0.5)	Wearing a Helmet The path is safe	2.2 (1.2)	0.6 (0.5)
Sawing wood	1.6 (1.0)	FUT	2 (0.9)	To hurt/cut oneself To reproduce this behaviour alone	−0.4 (1.6)	1.5 (1.0)	0.7 (0.5)	To be accompanied by an adult	1.1 (1.2)	0.3 (0.5)
Playing on the edge of a pond	2.2 (0.8)	NAT. MRD. FUN. SSS. MAB	1.6 (0.9)	To fall in the water To reproduce this behaviour alone	0.6 (1.4)	2.3 (0.7)	1.1 (0.4)	To be accompanied by an adult	2.4 (1.0)	0.8 (0.4)
Sword playing with wood	2.0 (0.8)	REO. FUN. MAB	1.2 (0.9)	To hurt oneself or somebody	0.8 (1.5)	2.7 (0.6)	1.0 (0.6)	In an attitude of play and not of quarrel	2.3 (1.0)	0.8 (0.4)
Playing in the woods	2.4 (0.7)	NAT. DAU. FUN. SCF. SSS. IFF. MRD	1.1 (0.9)	To get lost	1.3 (1.4)	2.5 (0.7)	1.1 (0.5)	Stay within sight of the child and return regularly	2.1 (1.1)	0.8 (0.4)
Playing in the rain	2.3 (0.8)	FUN. NAT. SCF. SSS	0.4 (0.7)	To get sick	1.9 (1.1)	2.7 (0.6)	1.2 (0.6)	To be well equipped	2.4 (1.0)	0.8 (0.4)
Petting a dog	1.9 (0.8)	DAN. MRD. SCF	1.6 (0.8)	The dog could bite Inappropriate attitude towards dog To believe that all dogs are nice	0.3 (1.4)	2.0 (0.9)	1.0 (0.4)	The owner of the dog is present and he/she holds the dog on a leash	2.6 (0.8)	0.9 (0.3)
Eating berries	2.0 (0.9)	NAT. SSS	1.3 (1)	Picking inedible berries	0.6 (1.7)	0.8 (1.0)	0.9 (0.5)	To check the picked berries	2.1 (1.2)	0.8 (0.4)
Running barefoot	2.4 (0.8)	SSS. NAT. FUN. MAB. IFF	0.9 (0.9)	To walk on a sharp object	1.5 (1.4)	2.7 (0.6)	1.3 (0.6)	In a protected and clean space	2.7 (0.8)	0.9 (0.3)

* Only the most frequently cited elements and those cited by at least 50% of parents are included in the table. (IFF) improve physical fitness; (MAB) Develop motor skills, agility, balance; (FUT) Develop a functional and/or utilitarian task; (REO) Develop relationships with others; (DAN) Develop learning to deal appropriately with the animal; (DAU) Developing autonomy; (MRD) knowing one’s limits and managing risks and dangers; (SCF) Developing self-confidence, taming one’s fears; (SSS) Discovering new sensations and stimulating one’s senses (touch, sight, kinesthesia, …); (NAT) Discover the environment/be in contact with nature; (FUN) Have fun.

**Table 4:** Pearson coefficients of correlation between child age, parent and child experiences, parental perceptions, and permission to play.

	CAge	CExp	PExp	PBen	PDang	PComp	PERM
CAge	1	0.40 **	0.21 **	0.09	−0.13 *	0.40 **	0.17 **
CExp		1	0.52 **	0.56 **	−0.34 **	0.67 **	0.50 **
PExp			1	0.42 **	−0.31 **	0.38 **	0.40 **
PBen				1	−0.55 **	0.66 **	0.75 **
PDang					1	−0.41 **	−0.60 **
PComp						1	0.58 **
PERM							1

Level of significance: * *p* < 0.01; ** *p* < 0.001.

**Table 5:** Linear regressions modelling the effects of child age (CAge), parent experience (PExp), and child experience (CExp) on parents’ representations: perceived benefits (PBen), perceived dangers (PDang), and perceived competence (PComp).

PDang	R = 0.37 R^2^ = 0.14 Adjusted R^2^ = 0.13 F(3,413) = 22.039 *p* < 0.001 SEE: 0.43766						
		b*	SE. (of b*)	b	SE. (of b)	t	*p*-value
	Intercept			2.00	0.09	21.36	<0.001
	CAge	0.01	0.05	0.00	0.02	0.12	0.91
	PExp	−0.24	0.06	−0.19	0.04	−4.26	<0.001
	CExp	−0.18	0.05	−0.40	0.12	−3.44	<0.001
PBen	R = 0.59 R^2^ = 0.35 Adjusted R^2^ = 0.35 F(3,413) = 75.104 *p* < 0.001 SEE: 0.41234						
		b*	SE. (of b*)	b	SE. (of b)	t	*p*-value
	Intercept			1.04	0.09	11.76	<0.001
	CAge	−0.16	0.04	−0.05	0.02	−3.60	<0.001
	PExp	0.17	0.05	0.42	0.11	3.77	<0.001
	CExp	0.53	0.05	0.44	0.04	10.70	<0.001
PComp	R = 0.69 R^2^ = 0.47 Adjusted R^2^ = 0.47 F(3,413) = 123.45 *p* < 0.001 SEE: 0.31338						
		b*	SE. (of b*)	b	SE. (of b)	t	*p*-value
	Intercept			0.98	0.07	14.58	<0.001
	CAge	0.15	0.04	0.05	0.01	3.92	<0.001
	PExp	0.05	0.04	0.09	0.08	1.12	0.26
	CExp	0.59	0.04	0.41	0.03	13.17	<0.001

b* = standardised beta coefficients of regression; b = beta coefficients of regression; SE = Standard error; SEE: Standard error of estimate.

**Table 6:** Linear regression modelling the effects of the explanatory variables and the PERM score.

	R = 0.81 R^2^ = 0.65 Adjusted R^2^ = 0.64 F(6,409) = 125.56 *p* < 0.001 SEE: 0.165					
	b*	Std. Err. (of b*)	b	Std. Err. (of b)	t	*p*-Value
Intercept			0.46	0.07	6.76	<0.001
CAge	0.04	0.03	0.01	0.01	1.09	0.28
PExp	0.06	0.04	0.06	0.05	1.31	0.19
CExp	0.02	0.04	0.01	0.02	0.38	0.71
PBen	0.52	0.05	0.28	0.02	11.33	<0.001
PDang	−0.27	0.04	−0.16	0.02	−7.67	<0.001
PComp	0.09	0.05	0.06	0.03	1.89	0.06

b* = standardised beta coefficients of regression; b = beta coefficients of regression; SE = Standard error; SEE: Standard error of estimate. The linear regression was performed on 416 subjects, after exclusion of an outlier.

## Data Availability

The data presented in this study are available on request from the corresponding author.

## Supplementary Materials

The following supporting information can be downloaded at: https://www.mdpi.com/article/10.3390/ijerph191811467/s1, File S1: pre-defined lists of answers for each situation and each of the sub-questions established by the researchers.
