# Supplementary material for: Parental Assessment of Benefits and of Dangers Determines Children’s Permission to Play Outdoors
Source: Int J Environ Res Public Health. 2022 Sep 12;19(18):11467. doi: 10.3390/ijerph191811467 (PMC9517668; doi:10.3390/ijerph191811467)
Supplement: Supplementary file 1 [file ijerph-19-11467-s001.zip › ijerph-1798366-supplementary.pdf]

Situation 1/10 : You are walking in the middle of nature and on the edge of the path there are rocks like on the picture. Your child wants to climb on them.

Q1b : What danger(s) are you most concerned about in this situation?

- He could slip and get hurt
- He may not know how to get down
- He may ruin his clothes
- He may repeat this behaviour without supervision (or develop a bad habit)
- Other

Q2b : What do you think is/are the benefit(s) of this type of situation for your child?

- Exercise and improve physical condition
- Develop motor skills, agility and balance
- Develop their autonomy
- Develop your relationship with others
- Know your limits better and manage risks and dangers
- Develop self-confidence, tame fears
- To discover new sensations and stimulate one's senses (touch, sight, kinesthesia, ...)
- Discover the environment/be in contact with nature
- To have fun
- Other

Q4b : Under what conditions would you allow your child to do this type of activity/situation?

- Be accompanied/assisted by an adult
- That he/she wears appropriate clothing
- That the rock is not too high (less than one meter)
- That the rock does not present any risks (slippery, sharp, ...)
- That he is older
- Other

Situation 2/10 : You are walking on a path with your child who has a balance bike like on the picture. There is a slight descent. He wants to be carried away by the speed without braking. There are no dangerous elements around.

Q1b : What danger(s) are you most concerned about in this situation?

- He could fall and/or injure himself
- He could walk away in an unsafe manner
- Could hit someone or something if they are not in control of their speed and/or trajectory
- Other

Q2b : What do you think is/are the benefit(s) of this type of situation for your child?

- Exercise and improve physical condition
- Develop motor skills, agility and balance
- Develop their autonomy
- Develop your relationship with others
- Know your limits better and manage risks and dangers

- Develop self-confidence, tame fears
- To discover new sensations and stimulate one's senses (touch, sight, kinesthesia, ...)
- Discover the environment/be in contact with nature
- To have fun
- Other

Q4b : Under what conditions would you allow your child to do this type of activity/situation?

- Be accompanied/assisted by an adult
- Wear a helmet
- He/she does not drive too fast
- That he/she manages his/her driving perfectly
- He/she does not stray too far
- That the path is safe
- That he is older
- Other

Situation 3/10 : You are sawing wood in your garden like in the picture. Your child asks you to try.

Q1b : What danger(s) are you most concerned about in this situation?

- He could hurt himself/cut himself
- Could hurt someone
- Could repeat the behaviour unsupervised (or develop a bad habit)
- Other

Q2b : What do you think is/are the benefit(s) of this type of situation for your child?

- Exercise and improve physical condition
- Develop motor skills, agility and balance
- Development of a functional and/or utilitarian task
- To develop one's autonomy
- Develop one's relationship with others
- To better understand one's limits and manage risks and dangers
- Develop self-confidence, tame fears
- To discover new sensations and stimulate one's senses (touch, sight, kinesthesia, ...)
- Discover the environment/be in contact with nature
- To have fun
- Other

Q4b : Under what conditions would you allow your child to do this type of activity/situation?

- That he is accompanied/assisted by an adult
- That he wears different protections (work gloves, mask, helmet ...)
- That it is a saw adapted for children
- That he has learned to handle comparable tools
- That he is older
- Other

Situation 4/10 : You are walking with your child. There is a shallow pond and he or she comes up to the edge to look at what's in the water, like in the picture.

Q1b : What danger(s) are you most concerned about in this situation?

- He could fall in the water
- He could drown
- He could be injured by rocks in the water
- Could get his clothes dirty
- May repeat the behavior without supervision (or develop a bad habit)
- Other

Q2b : What do you think is/are the benefit(s) of this type of situation for your child?

- Exercise and improve physical condition
- Develop motor skills, agility and balance
- Develop their autonomy
- Develop your relationship with others
- Know your limits better and manage risks and dangers
- Develop self-confidence, tame fears
- To discover new sensations and stimulate one's senses (touch, sight, kinesthesia, ...)
- Discover the environment/be in contact with nature
- To have fun
- Other

Q4b : Under what conditions would you allow your child to do this type of activity/situation?

- That it is supervised by an adult
- The weather should be nice
- That he/she knows how to swim
- That the water appears clean (transparent, free of visible waste, pollution)
- The water should not be too deep
- He/she is older
- Other

Situation 5/10 : You are walking in the woods with your child and friends who have children of the same age. They collect branches of the woods and start playing sword like in the picture..

Q1b : What danger(s) are you most concerned about in this situation?

- He could hurt himself and/or the other child (a piece of wood in the eye, in the belly, scratched...)
- He could hurt himself
- He could fall while running with the stick
- He could get dirty or ruin his clothes
- He/she could repeat this behavior without supervision (or develop a bad habit)
- Other

Q2b : What do you think is/are the benefit(s) of this type of situation for your child?

- Exercise and improve physical condition
- Develop motor skills, agility and balance
- Develop their autonomy
- Develop your relationship with others
- Know your limits better and manage risks and dangers
- Develop self-confidence, tame fears
- To discover new sensations and stimulate one's senses (touch, sight, kinesthesia, ...)
- Discover the environment/be in contact with nature
- To have fun
- Other

Q4b : Under what conditions would you allow your child to do this type of activity/situation?

- That they play fight with sticks that are not too big
- That they are in a playful attitude and not in a real quarrel
- That they are older
- Other

Situation 6/10 : You are having a picnic in the woods with friends and your child. During this break, your child wants to play freely in nature.

Q1b : What danger(s) are you most concerned about in this situation?

- He could get lost
- He could trip over rocks or tree roots and get hurt
- He could be attacked by an animal
- He could have a bad encounter
- He could put poisonous plants in his mouth
- Other

Q2b : What do you think is/are the benefit(s) of this type of situation for your child?

- Exercise and improve physical condition
- Develop motor skills, agility and balance
- Develop their autonomy
- Develop your relationship with others
- Know your limits better and manage risks and dangers
- Develop self-confidence, tame fears
- To discover new sensations and stimulate one's senses (touch, sight, kinesthesia, ...)
- Discover the environment/be in contact with nature
- To have fun
- Other

Q4b : Under what conditions would you allow your child to do this type of activity/situation?

- That he be accompanied by another child
- That it remains within sight, that it returns regularly
- That the wood does not present any danger
- That he is older
- Other

Situation 7/10 : It's raining outside and your child wants to go play in the garden like in the picture.

Q1b : What danger(s) are you most concerned about in this situation?

- He might get sick
- He could get cold
- He could slip and get hurt
- He could get his clothes dirty
- He/she might get the house dirty on the way home
- Other

Q2b : What do you think is/are the benefit(s) of this type of situation for your child?

- Exercise and improve physical condition
- Develop motor skills, agility and balance
- Develop their autonomy
- Develop your relationship with others
- Know your limits better and manage risks and dangers
- Develop self-confidence, tame fears
- To discover new sensations and stimulate one's senses (touch, sight, kinesthesia, ...)
- Discover the environment/be in contact with nature
- To have fun
- Other

Q4b : Under what conditions would you allow your child to do this type of activity/situation?

- That it rains moderately and that there is no risk of storm
- That he is well equipped (boots, coat, hat, ...)
- That it remains within sight
- That he changes immediately when he comes back home
- That the outside temperature is sufficient
- That he/she wears clothes that he/she can get dirty/damaged
- That he is not sick
- That he/she is older
- Other

Situation 8/10 : You are walking in a park and your child sees a dog and approaches it to pet it. The owner tells you that the dog is nice to children.

Q1b : What danger(s) are you most concerned about in this situation?

- The dog could bite him
- My child could get worms or germs
- He could have an inappropriate attitude towards the dog and provoke its aggression
- They may think all dogs are nice
- He/she may repeat this behavior without supervision (or develop a bad habit)
- Other

Q2b : What do you think is/are the benefit(s) of this type of situation for your child?

- Develop an appropriate attitude towards the animal
- Know your limits better and manage the risks and dangers in front of an animal
- Develop self-confidence, tame fears
- Discover new sensations and stimulate your senses (touch, sight, kinesthesia, ...)
- Discover the environment/be in contact with nature
- To have fun
- Other

Q4b : Under what conditions would you allow your child to do this type of activity/situation?

- That the owner of the dog is present and that he/she holds the dog on a leash
- That the dog seems friendly
- That he pats the dog on his back
- The dog owner should not stand in the dog's mouth
- That he is older
- Other

Situation 9/10 : You are walking with your child on a path. He sees some berries (blackberries) that are edible and starts picking them.

Q1b : What danger(s) are you most concerned about in this situation?

- He may pick inedible berries
- May pick berries too close to the ground that have been urinated on by animals
- He may have an allergic reaction
- May repeat this behavior without supervision (or develop a bad habit)
- Other

Q2b : What do you think is/are the benefit(s) of this type of situation for your child?

- Develop your autonomy
- Know your limits better and manage risks and dangers
- Develop self-confidence, tame fears
- Discover new sensations and stimulate your senses (touch, sight, kinesthesia, ...)
- Discover the environment/be in contact with nature
- To have fun
- Other

Q4b : Under what conditions would you allow your child to do this type of activity/situation?

- Have an adult check the berries they pick
- That they know how to recognize edible berries
- That the berries are picked at a certain height (above the knee)
- Other

Situation 10/10 : You are in a park with your child and he wants to take off his shoes to run barefoot.

Q1b : What danger(s) are you most concerned about in this situation?

- He could get his feet dirty (mud, earth, animal excrements...)

- He could step on a sharp object (broken glass, cane, ...)
- He could step on a stinging insect (wasp, ants,...)
- He could have an allergic reaction
- Other

Q2b : What do you think is/are the benefit(s) of this type of situation for your child?

- Exercise and improve physical condition
- Develop motor skills, agility and balance
- Develop their autonomy
- Develop your relationship with others
- Know your limits better and manage risks and dangers
- Develop self-confidence, tame fears
- To discover new sensations and stimulate one's senses (touch, sight, kinesthesia, ...)
- Discover the environment/be in contact with nature
- To have fun
- Other

Q4b : Under what conditions would you allow your child to do this type of activity/situation?

- That it is in a protected and clean space (private park, garden)
- That it is not too far away
- That there are no wasps nearby
- Other
